# Epidemiological trends of infant mortality related to premature rupture of membranes: U.S. 1999–2023

**DOI:** 10.3389/fpubh.2025.1715138

**Published:** 2026-01-12

**Authors:** Lillian Eason, Isabella Zent, Mariah Brown, Abubakar Tauseef, Vikram Murugan

**Affiliations:** 1 Creighton University School of Medicine, Omaha, NE, United States; 2 Department of Internal Medicine, Creighton University, Omaha, NE, United States

**Keywords:** demographics, health disparities, infant mortality, neonatal outcomes, perinatal epidemiology CDC WONDER, premature rupture of membrane (PROM), United States

## Abstract

**Introduction:**

Premature rupture of membranes (PROM) complicates 8% of pregnancies and accounts for nearly one-third of preterm labor. While prior studies have examined clinical management and maternal risk factors, national data on PROM-related infant mortality remains scarce. This study investigates demographic trends in PROM-related infant mortality in the U.S. from 1999 to 2023.

**Methods:**

The CDC WONDER database was utilized to identify U.S. crude mortality rates of newborns affected by PROM (ICD-10 code P01.1). Demographic variables were analyzed through the CDC Joinpoint Regression program to evaluate temporal trends. This study used publicly available, de-identified data and is IRB-exempt.

**Results:**

A total of 27,184 PROM-related infant deaths were identified from 1999 to 2023. Mortality rates rose significantly from 1999 to 2002 (APC = 5.09), increased modestly through 2013 (APC = 0.69), then declined through 2023 (APC = −3.66). Black or African American infants consistently had higher mortality across gender and region. In urban areas, mortality increased significantly in large central metros (APC = 2.32) and fringe metros (APC = 1.45) until 2012, then declined through 2023 (APC = −2.93; −1.45). Rural areas showed a gradual decline from 1999 to 2021 (APC = −1.35).

**Conclusion:**

While PROM-related infant mortality has declined since the early 2000s, disparities persist across race and geography. Black or African American infants continue to experience higher mortality rates, mirroring broader obstetric disparities. Urban and rural centers also demonstrated distinct temporal patterns. Further research is needed to clarify these trends and risk factors between these sub-populations.

## Introduction

Premature rupture of membranes (PROM) is defined as the rupture of fetal membranes before the onset of labor, resulting in the leakage of amniotic fluid (1, 2). PROM is generally considered at term if it occurs after 37 weeks of gestation, whereas Preterm Premature Rupture of Membranes (PPROM) is considered rupture before 37 weeks. PROM is estimated to complicate 8% of pregnancies, whereas PPROM is estimated to occur in 1–3% of pregnancies and cause roughly one-third of all spontaneous preterm deliveries (3–6).

The etiologies of PROM are multifactorial and include, but are not limited to, uterine distension from polyhydramnios or multifetal pregnancies, alterations in metalloprotein expression in fetal membrane including inflammasomes, maternal connective tissue disorders, intraamniotic infections, short cervical length, antepartum vaginal bleeding, low body mass index, low socioeconomic status, cigarette smoking and illicit drug use (1, 7–10). Additionally, there is a considerable risk of recurrence of PPROM in future pregnancies, ranging from 10 to 32%, with the most common complication in subsequent pregnancies being preterm birth (5).

PROM carries both maternal and neonatal risks. Maternal complications include intrauterine infection, sepsis, placental abruption, hemorrhage, and death (4). Neonatal and fetal risks include, but are not limited to, neonatal sepsis, premature birth and associated complications, prolonged oligohydramnios with associated pulmonary hypoplasia and limb contractures, and respiratory distress syndrome (4, 5, 11). The infant prognosis is dependent on the gestational age at which rupture of membranes occurs, with PROM at term the most favorable neonatal survival rate of 80 to 90%, followed by PPROM after 22 weeks of gestation (58%), and finally PPROM before 22 weeks (14–22%) (4, 11–13). Additionally, the latency period, or the time between rupture of membranes and onset of labor and delivery, affects infant mortality. A shorter latency period for PPROM before 26 to 30 weeks is associated with higher infant mortality rates. In contrast, a shorter latency period with PPROM after 28 to 30 weeks of gestation is associated with lower infant mortality (14, 15) Majority of PROM-related deaths occur in the neonatal period, defined at less than 28 days old, with rare occurrences into infancy (16).

The risk of complications and mortality in both maternal and fetal health makes PROM important to recognize early and treat appropriately. Treatment varies with gestational age but generally includes expectant management until 37 weeks (5, 17, 18). Expectant management includes antibiotics for Group B Streptococci, corticosteroids if between 23 and 34 weeks of gestation, tocolytics if labor needs to be delayed, and magnesium sulfate for neuroprotection when less than 32 weeks of gestation (5, 17). If infection is suspected, additional broad-spectrum antibiotics are given (5, 17).

There is limited research investigating risk factors of PROM and its related mortality. Current literature analyzing risk factors and trends in demographics for PROM focuses on maternal characteristics, including parity, education, occupation, diabetes, blood pressure, cervical length, and body mass index (19–21). Additionally, obstetric history, including previous abortions, preterm deliveries, prior PROM, cesarean sections, gestational hypertension, diabetes mellitus, infections, and delivery or neonatal complications and outcomes, is taken into consideration (19–21).

Additionally, several hospital-based retrospective cohort studies have examined PROM. Endale et al. (2016) (45) conducted a retrospective cross-sectional study from 2011 to 2013 of women diagnosed with PROM at Mizan-Aman General Hospital, Ethiopia. The study collected socio-demographic and obstetric profiles of patients, including their residence status, urban or rural. Another retrospective chart review study by Rattan and Ramnarain (19) collected maternal and neonatal parameters from women diagnosed with PPROM, defined as less than 34 weeks at General Justice Gizenga Mpanza Hospital, South Africa, in 2018. The study collected parameters such as maternal factors previously mentioned, neonatal outcomes, and obstetric history and complications. Although these studies analyze risk factors and determinants of PROM, they mainly focus on obstetric history and risk factors. Infant demographic factors, such as race, region, gender, and their intersectionality, have yet to be studied.

This study aims to address the gaps in the literature concerning the demographic factors and trends of infant mortality among women diagnosed with PROM. This study does not distinguish between PROM and PPROM, and therefore analysis of mortality trends in this study views both diagnoses as a single entity under PROM. The Center for Disease Control and Prevention’s Wide-ranging Online Data for Epidemiological Research (CDC WONDER) database is used to analyze data related to race, gender, region, and urbanization, and their intersections, in women diagnosed with PROM from 1999 to 2023. Utilizing this data, we provide a temporal evaluation of trends in infant mortality related to PROM and evaluate disparities between demographic groups within the study population.

## Materials and methods

CDC WONDER was used to identify infant deaths within the United States, with premature rupture of membranes listed as one of the causes of death between 1999 and 2023 (22). Multiple-cause mortality data were examined using the International Classification of Diseases, 10th Revision (ICD-10) code 130 (Infants) P01.1: Newborn affected by premature rupture of membranes. ICD-10 code P01.1 includes all newborns affected by premature rupture of membranes but does not account for gestational age at time of rupture, therefore infants affected by PPROM and PROM cannot be distinguished (22, 23). This dataset includes liveborn newborns affected by PROM and does not account for fetal deaths resulting from PROM. This coding approach is consistent with current literature investigating infant mortality trends in the United States population (24, 25). Since CDC WONDER does not allow race-stratification by specific infant age groups, the less than one-year-old age group was utilized for all factors examining race. For all other demographic variables, infant age groups were used. CDC WONDER defines infant as less than one-year old, therefore the term infant is utilized for all data points whether representing neonatal period or other up to a year old. This study was exempt from institutional review board approval as CDC WONDER contains publicly available, de-identified data.

This analysis investigates race/ethnicity (Asian or Pacific Islander non-Hispanic, Black or African American non-Hispanic, White non-Hispanic, and Hispanic or Latino), gender (male or female), census region at time of death (Northeast, Midwest, South, and West), and urban–rural classification based on the 2013 National Center for Health Statistics Urban–Rural Classification scheme for countries.

Infant mortality related to PROM was assessed by calculating crude mortality rates, calculated by CDC WONDER as the number of PROM-related infant deaths divided by the number of registered live births in the U.S. during this time. Only crude mortality rates were used because age-adjusted mortality rates (AAMRs) cannot be calculated for infant subgroups, as they fall within a singular age population.

Trend analysis was conducted via the Joinpoint Regression Program (version 5.4.0), similar to previous CDC WONDER database studies, to form Joinpoint Regression models (24, 26, 27).

Joinpoint regression fits segmented log-linear models to determine statistically significant changes in trends, also known as joinpoints, via the Monte Carlo permutation method. Annual percent changes (APCs) and average annual percentage change (AAPC) were calculated through this program based on these segments.

## Results

### Overall

**Figure 1:**
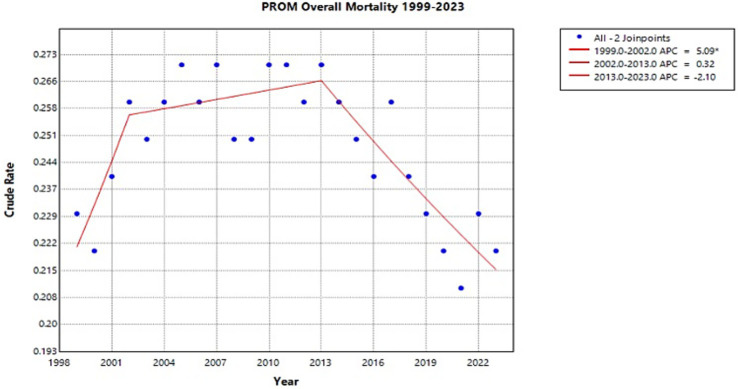
Joinpoint model of newborns affected by premature rupture of membranes crude mortality rate per 100,000 live births, 1999–2023 (*APC significant).

From 1999 to 2023, there were 27,184 infant deaths related to PROM in the United States (Joinpoint Figure 1). The crude rate significantly increased from 0.23 to 0.26 between 1999 and 2002, with an APC of 5.09* (95% CI 0.41 to 14.65) (Joinpoint Figure 1). Between 2002 and 2013, the crude rate rose slightly to 0.27, although at a nonsignificant APC of 0.32 (95% CI −5.71–1.61) (Joinpoint Figure 1). After 2013, there was a downward trend to a crude rate of 0.22 in 2023, corresponding with an APC of −2.10 (95% CI −7.20–1.64) (Joinpoint Figure 1). The overall data from 1999 to 2023 has a nonsignificant AAPC of −0.12 (−0.77 to 0.49) (Joinpoint Figure 1).

### Sex

**Figure 2:**
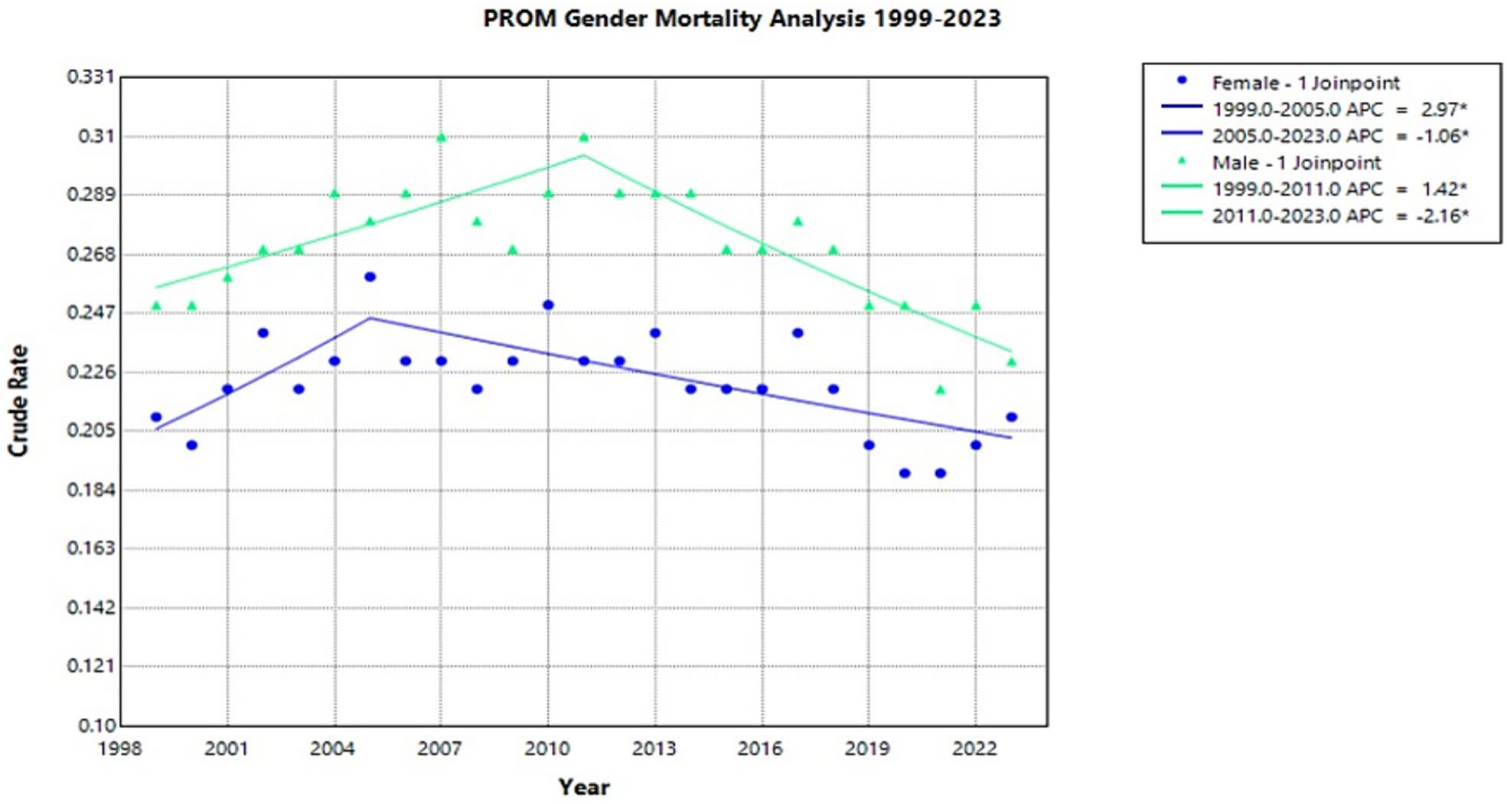
Joinpoint model of newborns affected by premature rupture of membranes crude mortality rate per 100,000 live births by gender, 1999–2023 (*APC significant).

From 1999 to 2023, PROM-related infant mortality showed no statistically significant overall trend in either male or female groups. Male infants consistently experienced higher crude mortality rates than female infants throughout the study period. The female crude rate significantly increased from 1999 to 2005, with an APC of 2.97* (95% CI 0.14 to 13.11), followed by a significant decrease of −1.06* (95% CI −3.20 to −0.53) from 2005 to 2023 (Joinpoint Figure 2). Male infant mortality showed a similar trend with a significant increase from 1999 to 2011, with an APC of 1.42* (95% CI 0.70–2.45), followed by a significant decrease of −2.16* (95% CI −3.20 to −1.45) from 2011 to 2023(Joinpoint Figure 2).

### Race

**Figure 3:**
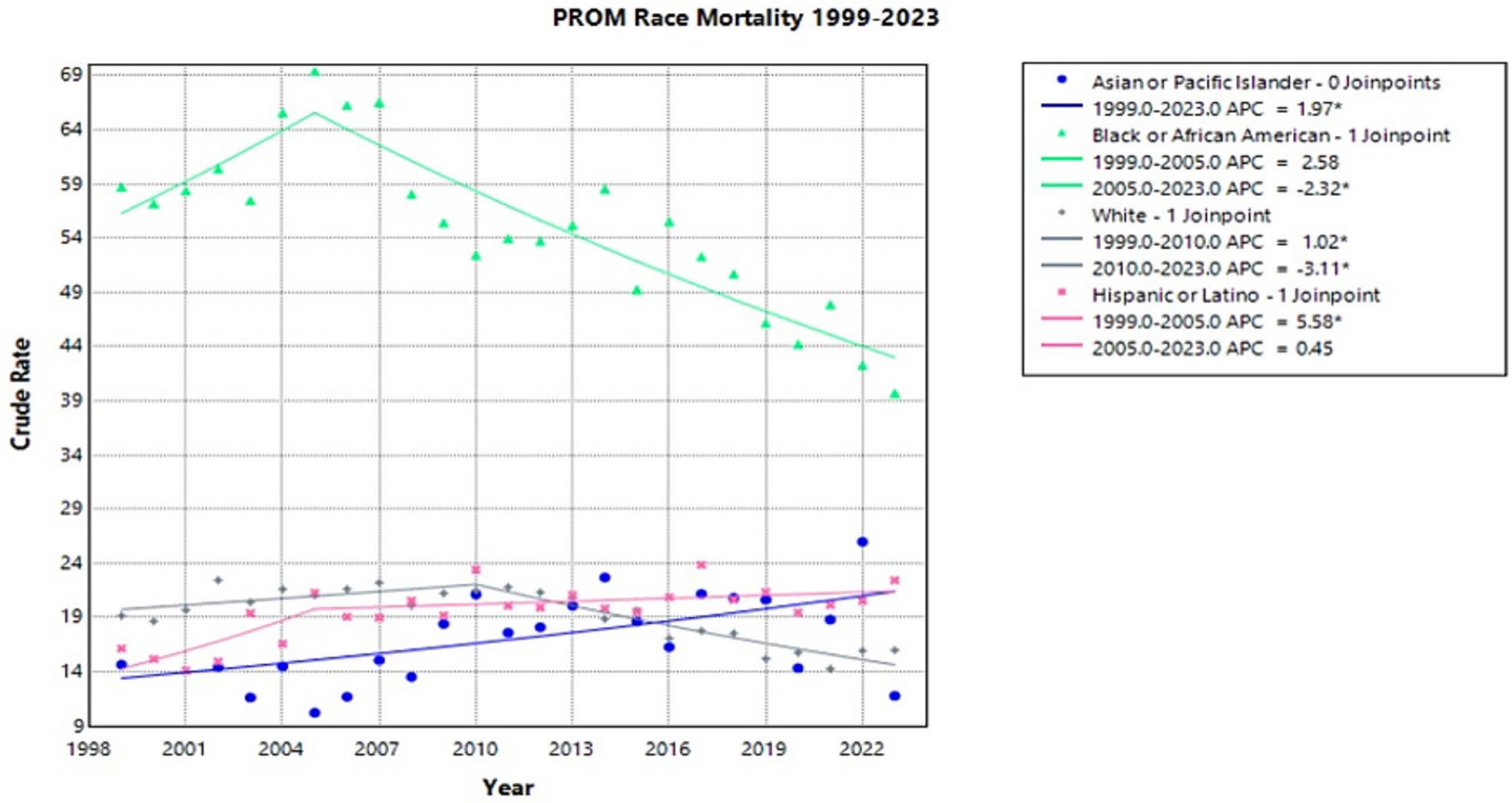
Joinpoint model of newborns affected by premature rupture of membranes crude mortality rate per 100,000 live births by race, 1999–2023 (*APC significant).

From 1999 to 2023, there were 8,146 PROM-related deaths among Black or African American infants. From 1999 to 2005, there was an increase in crude rate from 58.73 to 69.41 with an APC of 2.58 (95% CI −0.44 to 12.31)(Joinpoint Figure 3). This was followed by a decrease in crude rate from 2005 to 2023 with an APC of −2.32* (95% CI −3.79 to −1.71) (Joinpoint Figure 3). Throughout the study period, Black or African American infants had consistently higher crude mortality rates than any other racial group.

There were 857 PROM-related deaths in the Asian American or Pacific Islander population between 1999 and 2023. During this time, there was a significant increase in crude mortality rates with an APC of 1.97* (95% CI 0.57–3.59) (Joinpoint Figure 3). The crude rate rose from 14.66 in 1999 to 25.99 in 2022, with a nonsignificant decrease to 11.77 in 2023. Asian American or Pacific Islanders had no significant decrease in crude rate during the study period.

Among the White population, there were 10,106 PROM-related deaths from 1999 to 2023 (Joinpoint Figure 3). From 1999 to 2010, there was a significant increase in crude rate from 19.17 to 21.51 with an APC of 1.02* (95% CI 0.06–2.43) (Joinpoint Figure 3). This was followed by a significant decrease from 2010 to 2023, with the crude rate decreasing to 15.99 with an APC of −3.11* (95% CI −4.34 to −2.31) (Joinpoint Figure 3).

In the Hispanic population, 4,805 PROM-related deaths were reported during the study period (Joinpoint Figure 3). From 1999 to 2005, there was a significant increase in crude rate from 16.14 to 21.29 with an APC of 5.58* (95% CI 1.74–21.10) (Joinpoint Figure 3). No significant decline was observed in PROM-related mortality in this population following 2005, and the data appear to plateau at this time. From 2004 to 2023, the crude rate decreased to 22.44 with an APC of 0.45 (95% CI −2.04 to 1.12) (Joinpoint Figure 3).

### Sex-race

**Figure 4:**
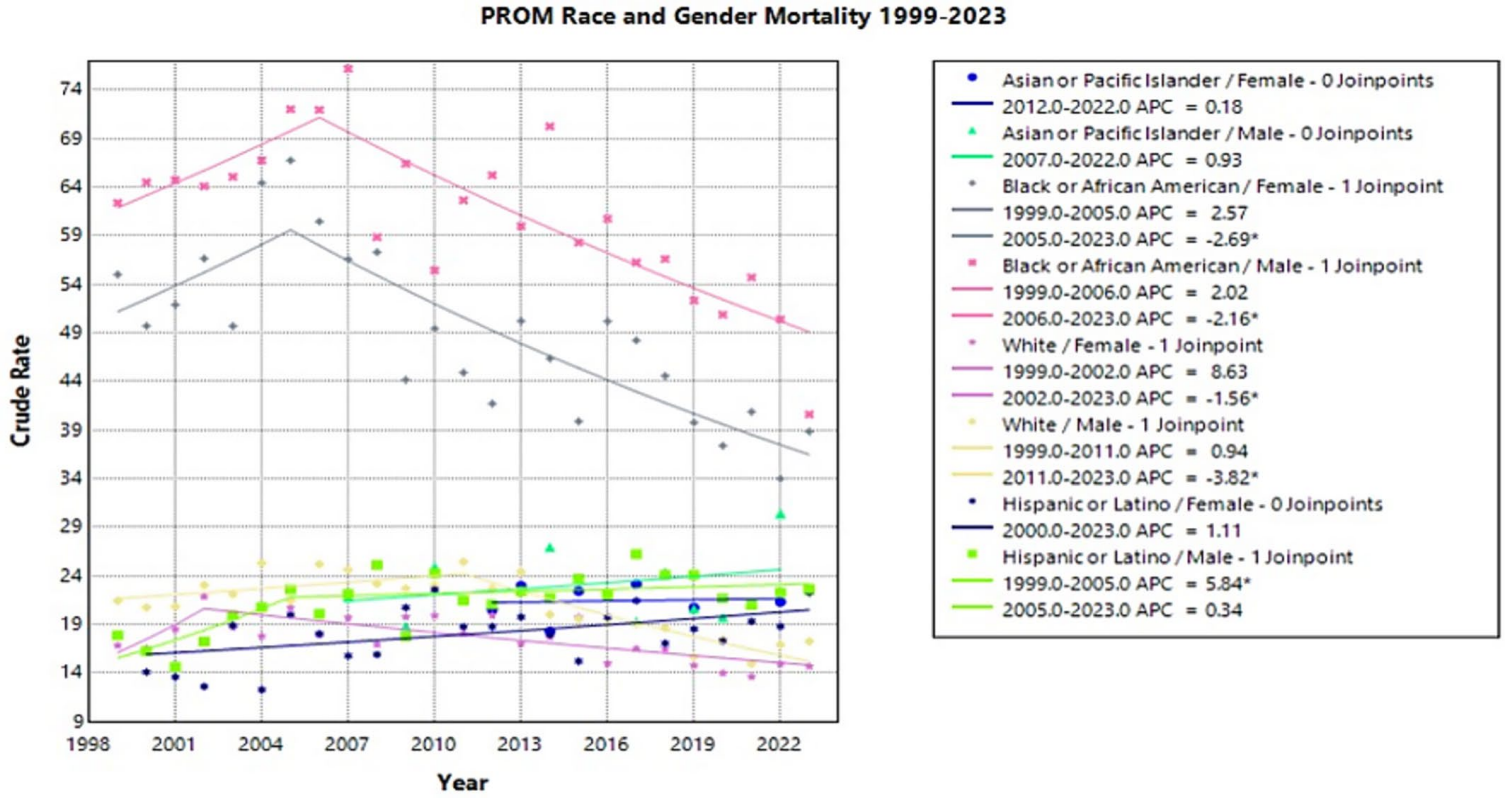
Joinpoint model of newborns affected by premature rupture of membranes crude mortality rate per 100,000 live births by race and gender, 1999–2023 (*APC significant).

Of the analyzed race by sex data, the Hispanic/Latino male population experienced the most significant change in crude rate with an APC of 5.84* (95% CI 1.69–24.61) from 1999 to 2005 (Joinpoint Figure 4). This upward trend was followed by a nonsignificant plateau from 2005 to 2023 with an APC of 0.34 (95% CI −2.58–1.06) (Joinpoint Figure 4). Overall, from 1999 to 2023, the AAPC for Hispanic/Latino males was 1.69* (95% CI 0.65–2.92), which was the highest AAPC seen across race and gender (Joinpoint Figure 4).

The Black or African American male and female populations experienced a similar downward trend in PROM-related crude mortality rate. From 2005 to 2023, the female population showed a significant APC of −2.69* (95% CI −6.86 to −1.91) while the male population showed a similar significant APC of −2.16* (95% CI −3.94 to −1.49) from 2006 to 2023 (Joinpoint Figure 4). Overall, female Black or African American infants had a significant AAPC from 1999 to 2023 of −1.40* (95% CI −2.51 to −0.40) while the male population showed a significant AAPC of −0.96* (95% CI −1.69 to −0.26) across the same time frame (Joinpoint Figure 4). Notably, Black or African American male and female populations experienced consistently higher crude mortality rates compared to all other racial groups throughout the study period.

While the white female population experienced a significant decrease in crude rate from 2002 to 2023, with an APC of −1.56* (95% CI −9.37 to −0.95), the white male population did not experience a significant decrease until 2011, with an APC of −3.82* (95% CI −6.05 to −2.59) from 2011 to 2023 (Joinpoint Figure 4). From 1999 to 2002, the White female population showed a nonsignificant increase, while White males had a nonsignificant plateau from 1999 to 2011, with APCs of 8.63 (95% CI −0.82–33.09) and 0.940 (95% CI −0.20–2.82), respectively (Joinpoint Figure 4). During the entire study period, the white male population had an AAPC of −1.47* (95% CI −2.15 to −0.88) (Joinpoint Figure 4).

### Region

**Figure 5:**
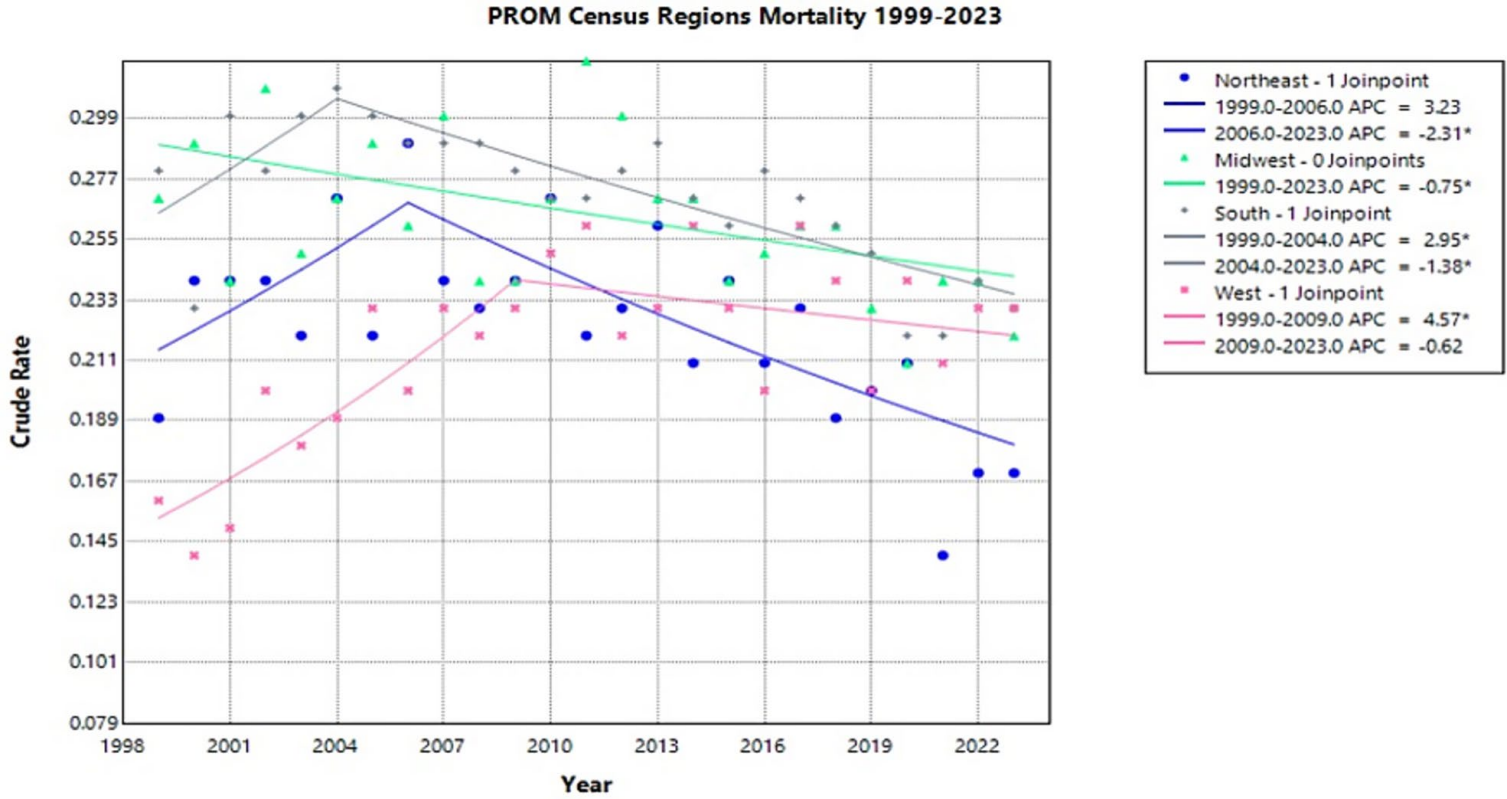
Joinpoint model of newborns affected by premature rupture of membranes crude mortality rate per 100,000 live births by census region, 1999–2023 (*APC significant).

From 2006 to 2023, the Northeast experienced a significant decrease in crude rate from 0.29 to 0.17 with an APC of −2.31* (95% CI −16.02 to −1.25) (Joinpoint Figure 5). From 1999 to 2023, the Midwest experienced a significant decrease in crude rate from 0.27 to 0.22 with an APC of −0.75* (95% CI −1.39 to −0.14) (Joinpoint Figure 5). Interestingly, from 1999 to 2004, the South experienced a significant increase in crude rate from 0.28 to 0.31 with an APC of 2.95* (95% CI 0.11–10.20) (Joinpoint Figure 5). This is followed by a significant decrease from 2004 to 2023, where the crude rate decreased to 0.23 with an APC of −1.38* (95% CI −2.26 to −0.98) (Joinpoint Figure 5). Similarly, the West from 1999 to 2009 experienced a significant increase in crude rate from 0.16 to 0.23 with an APC of 4.57* (95% CI 2.75–9.92) (Joinpoint Figure 5). However, the West did not have a significant decrease in crude rate following this timeframe. From 2009 to 2023, the crude rate decreased to 0.23 with an APC of −0.62 (95% CI −3.61–0.77) (Joinpoint Figure 5).

### Region-Gender

**Figure 6:**
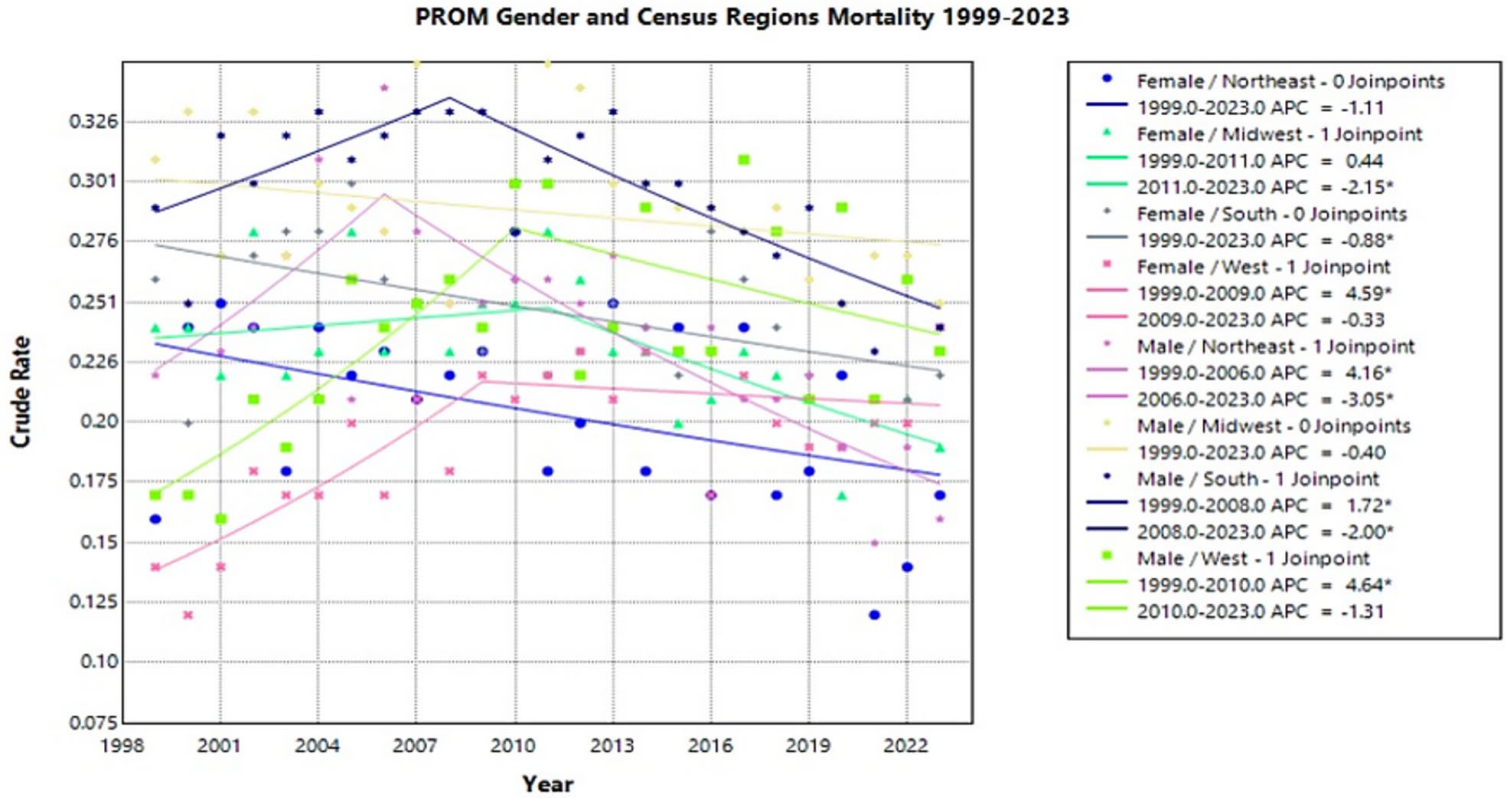
Joinpoint model of newborns affected by premature rupture of membranes crude mortality rate per 100,000 live births by gender and census region, 1999–2023 (*APC significant).

Of the analyzed data from region by gender, both male and female populations in the West experienced the largest change in crude rates. From 1999 to 2009, the females in the West had an APC of 4.59* (95% CI 2.04–21.33) while males in the West had an APC of 4.64* (95% CI 1.90–24.48) from 1999 to 2010 (Joinpoint Figure 6).

This was followed by males in the Northeast who had an APC of 4.16* (95% CI 0.65–17.43) from 1999 to 2006, followed by an APC of −3.05* (95% CI −5.32 to −1.96) from 2006 to 2023 (Joinpoint Figure 6). The females in the Northeast did not experience a significant change in crude rate from 1999 to 2023.

In the South, the female population had an overall significant decrease from 1999 to 2023 with an APC of −0.88* (95% CI −1.49 to −0.33) (Joinpoint Figure 6). Southern males had a significant increase from 1999 to 2008, followed by a significant decrease from 2008 to 2023 with an APC of 1.72* (95% CI 0.39–4.21) and −2.00* (95% CI −3.07 to −1.35), respectively (Joinpoint Figure 6).

In the Midwest, the female population had a significant decrease in crude rate from 2011 to 2023 with an APC of −2.15* (95% CI −9.42 to −1.02) following a nonsignificant plateau (Joinpoint Figure 6). Midwestern males’ mortality rates remained unchanged from 1999 to 2023.

Overall, where most regions showed a similar trend of an increase to peak crude rate followed by a steady decrease to 2023, females in the Northeast and South, and males in the Midwest show only a steady decline with no peak from 1999 to 2023.

### Region-Race

**Figure 7:**
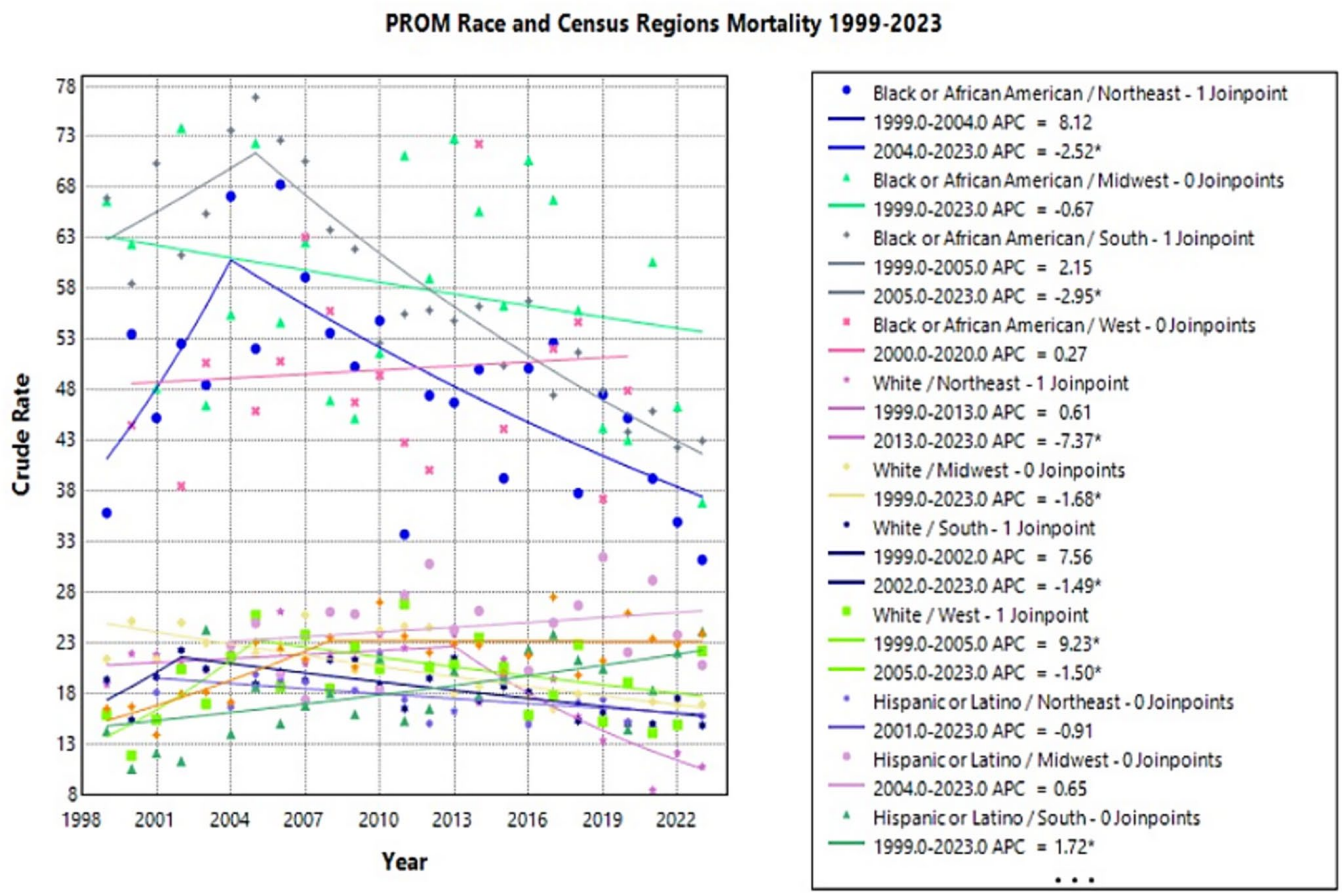
Joinpoint model of newborns affected by premature rupture of membranes crude mortality rate per 100,000 live births by race and census region, 1999–2023 (*APC significant).

Of the data analyzed from region by race, the white population in the West experienced the greatest change in crude rates. This group had a significant increase from 1999 to 2005, with an APC of 9.23* (95% CI 0.97–45.32) (Joinpoint Figure 7). This was followed by a significant decrease from 2005 to 2023 with an APC of −1.50* (95% CI −12.74 to −0.03) (Joinpoint Figure 7). The Hispanic or Latino population in the West had an APC of 4.75* (95% CI 2.22–16.13) from 1999 to 2008, which was followed by a nonsignificant plateau (Joinpoint Figure 7). The Black or African American population in the West did not undergo significant changes in crude rate from 1999 to 2023.

In the Midwest, the White population had a significant decrease from 1999 to 2023, with an APC of −1.68* (95% CI −2.38 to −1.03) (Joinpoint Figure 7). The Black or African American and Hispanic or Latino populations did not experience significant changes in crude rate from 1999 to 2023.

The White population in the Northeast underwent the most significant decrease in crude rate with an APC of −7.37* (95% CI −11.51 to −5.07) from 2013 to 2023 (Joinpoint Figure 7). The Black or African American population had a significant decrease from 2004 to 2023 with an APC of −2.52* (95% CI −10.59 to −1.43) (Joinpoint Figure 7).

In the South, both the White and Black of African American populations experienced significant decreases in crude rates. The White population had an APC of −1.49* (95% CI −10.82 to −0.82) from 2002 to 2023, while the Black or African American group had an APC of −2.95* (95% CI −4.13 to −2.36) from 2005 to 2023 (Joinpoint Figure 7). From 1999 to 2023, the Hispanic or Latino population had a significant increase with an APC of 1.72* (95% CI 0.62–3.00) (Joinpoint Figure 7). Notably, there was an observed trend of Black or African American populations experiencing higher overall crude rates in every region compared to all other races.

### Urban/rural

**Figure 8:**
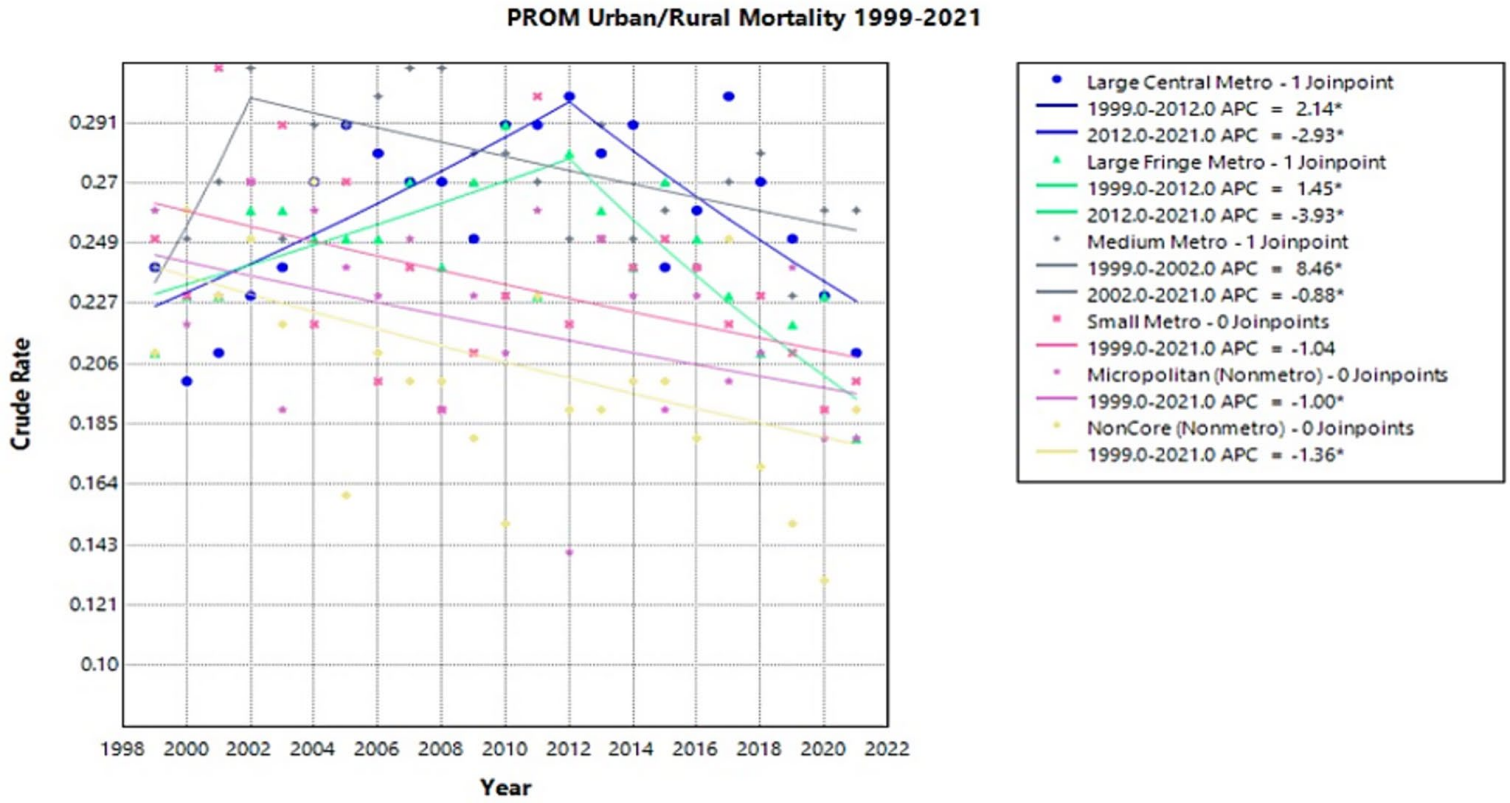
Joinpoint model of newborns affected by premature rupture of membranes crude mortality rate per 100,000 live births by urban/rural, 1999–2023 (*APC significant).

In large central metro areas, there was a significant increase in crude rate from 1999 to 2012 with an APC of 2.14* (95% CI 1.01–4.55) (Joinpoint Figure 8). This was followed by a significant decrease from 2012 to 2021 with an APC of −2.93* (95% CI −7.62 to −0.94) (Joinpoint Figure 8). Large fringe metro regions similarly had an increase in crude rate from 1999 to 2012 with an APC of 1.45* (95% CI 0.42–3.19), then a decrease from 2012 to 2021 with an APC of −3.93* (95% CI −6.31 to −2.44) (Joinpoint Figure 8). Medium metro areas also had a significant increase with an APC of 8.46* (95% CI 0.96–23.10) from 1999 to 2002, followed by a decrease from 2002 to 2021 with an APC of −0.88* (95% CI −1.902 to −0.40) (Joinpoint Figure 8). In micropolitan (nonmetro) areas, there was a significant decrease in crude rate with an APC of −1.00* (95% CI −2.03 to −0.01) from 1999 to 2021 (Joinpoint Figure 8). Noncore (nonmetro) areas showed a significant decrease from 1999 to 2021 with an APC of −1.36* (95% CI −2.59 to −0.29) (Joinpoint Figure 8). Small metro regions did not experience significant changes in crude rate.

### Gender – urban/rural

**Figure 9:**
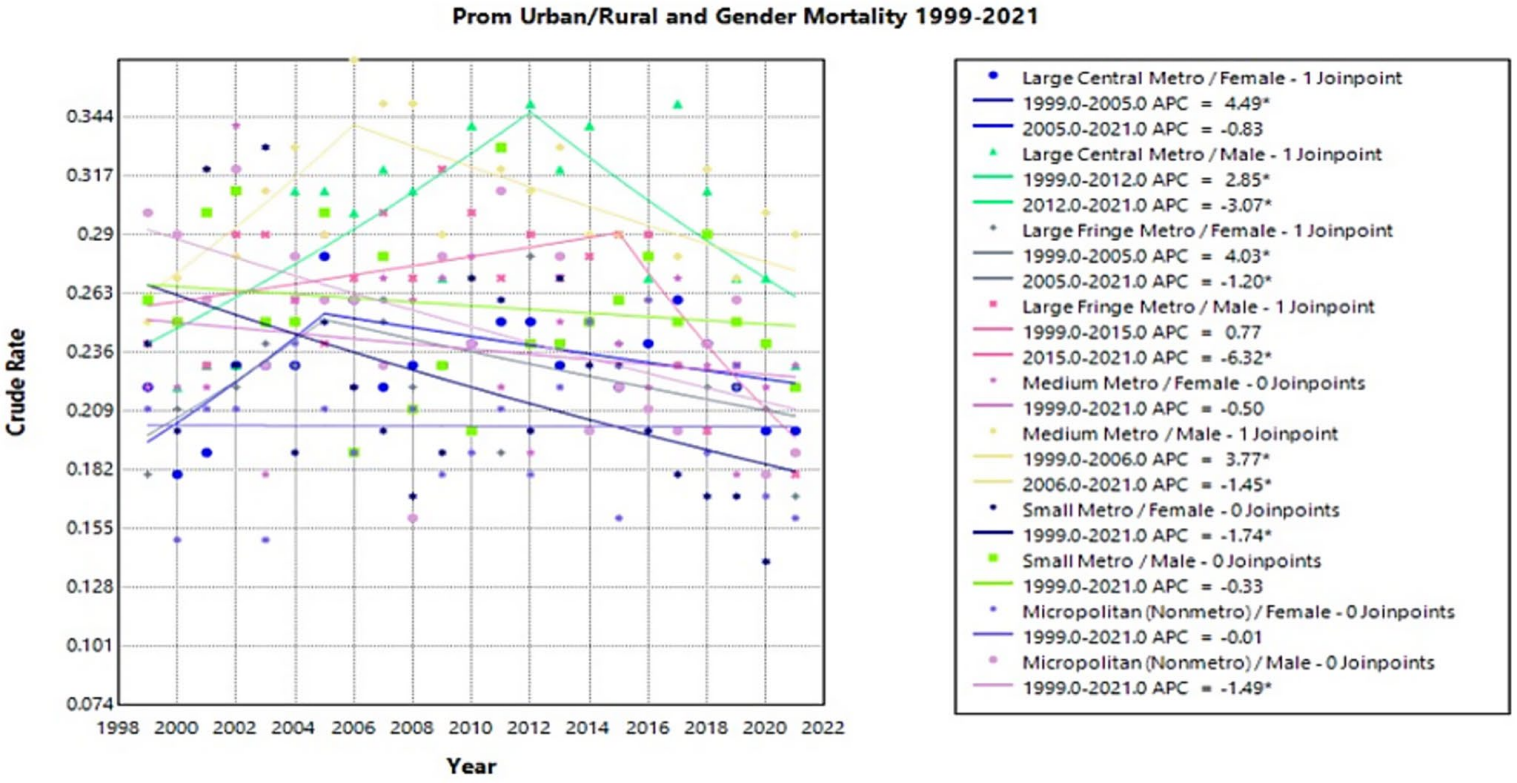
Joinpoint model of newborns affected by premature rupture of membranes crude mortality rate per 100,000 live births by urban/rural and gender, 1999–2023 (*APC significant).

Females in large central metro areas had a significant increase in crude rate from 1999 to 2005 with an APC of 4.49* (95% CI 0.54–19.43) (Joinpoint Figure 9). From 1999 to 2012, males in this region had a significant increase with an APC of 2.85* (95% CI 1.40–7.56) followed by a decrease and an APC of −3.07* (95% CI −10.08 to −0.52) from 2012 to 2021 (Joinpoint Figure 9).

In large fringe metro regions, females experienced a significant increase in crude rate from 1999 to 2005 with an APC of 4.03* (95% CI 0.17–21.39) (Joinpoint Figure 9). From 2005 to 2021, females then had a decrease with an APC of −1.20* (95% CI −8.03 to −0.27) (Joinpoint Figure 9). Males in this region had a significant decrease from 2015 to 2021 with an APC of −6.32* (95% CI −16.06 to −2.56) (Joinpoint Figure 9).

In medium metro areas, males had a significant increase in crude rate with an APC of 3.77* (95% CI 0.98–13.28) from 1999 to 2006, followed by a decrease with an APC of −1.45* (95% CI −4.19 to −0.52) from 2006 to 2021 (Joinpoint Figure 9).

From 1999 to 2021, females in small metro areas experienced a significant decrease with an APC of −1.74* (95% CI −3.36 to −0.26) (Joinpoint Figure 9). In micropolitan (nonmetro) areas, males had a significant decrease from 1999 to 2021 with an APC of −1.49* (95% CI −2.59 to −0.46) (Joinpoint Figure 9).

## Discussion

This study analyzed infant mortality trends associated with PROM from 1999 to 2023. The overall crude mortality rate increased until 2013, after which it declined until 2023 (Figure 1). The most notable increase occurred between 1999 and 2002 (Figure 1). As PROM is estimated to account for one-third of preterm births, analyzing these trends together provides context for understanding their interrelated impact on maternal and neonatal outcomes (5, 28). The early 2000s rise in infant mortality coincides with an estimated 30% increase in infants born prematurely between 1980 and 2000 (6). As this study is limited to 1999 to 2023, the trends prior to 1999 cannot be compared to this timeframe. However, we hypothesize that the increased premature birth rate correlates with the rise in infant mortality prior to 2002 seen in this analysis.

Along with this increase in preterm birth rates, advances in neonatology were improving survival for premature infants. Although the age of viability is controversial and considered a clinical judgment, in the 1960s it was thought to be around 28 weeks, but by the 1990s, around 50% of babies born at 24 weeks survived in the neonatal intensive care unit (NICU) (29, 30). Due to NICU advancements such as FDA approval of surfactant, modern incubators, respiratory ventilators, and neonatal nutrition, a 1-kg infant born in 2000 had a 95% probability of survival compared to 95% risk of mortality in 1960 (29). Additionally, a comparison study between eleven U.S. centers found a 6% increase in survival of infants born at 22 to 24 weeks gestation between a 2000 to 2003 cohort and 2008 to 2011 cohort (31). This increase in survival rate corresponds with the relative plateau of infant mortality rates between 2002 and 2013 and the ultimate decline of rates found in this study.

The decline in infant mortality rates after 2013 may be related to the expansion of Medicaid under the Affordable Care Act (ACA) in 2010 and the Children’s Health Insurance Program (CHIP) Reauthorization Act of 2009. Given that Medicaid has been estimated to cover roughly 45% of all U.S. births, its expansion would have a substantial impact (32, 33). The ACA mandated that insurance companies cover maternity and neonatal care, including prenatal care, labor and delivery, and postpartum care without changing costs due to pregnancy status. It also expanded coverage by raising the threshold income needed to qualify. Additionally, Medicaid and CHIP expanded coverage of pregnant women and children without the previous five-year residency requirement, opening coverage for women who immigrated to the U.S. (34) Prior to ACA initiatives, 1 in 4 women of reproductive age and 1 in 8 pregnant women reported being uninsured (35). Furthermore, low-income women who qualified for pregnancy-related Medicaid were uninsured 12 months before delivery and 6 months following birth due to the temporary coverage mandates prior to ACA (35). Having temporary coverage for women of reproductive age may create a discontinuity of care that leaves maternal health concerns unaddressed such as hypertension and diabetes mellitus which were found to be among the most important risk factors for PROM associated with neonatal and maternal complications (21, 35). An observational study comparing pre-policy and post-policy periods from 2010 to 2016, found a reduction of 11% of women of reproductive age not receiving or delaying care because of cost and a 14% decrease of women not having a usual source of care (35). Therefore, we hypothesize that by removing co-pays, mandating care, and increasing coverage, more women would have access to antenatal care, potentially leading to better outcomes (35).

An observational study of births from 2011 to 2016 found that states that implemented the Medicaid expansion saw significantly reduced preterm births and low birth weights for Black or African American women (36). Another study found a 14.5% decline in overall infant mortality in Black or African American infants in Medicaid expansion states, which was more than twice what was seen in non-Medicaid states (32). This corresponds with the significant decline in PROM-related mortality in the infants of Black or African American women seen in this study. The continuity of care that Medicaid expansion provided allowed for women to have access to contraceptive services, prenatal care, maternal chronic disease surveillance, and mental health management regardless of pregnancy status, which in turn may have reduced the effect of comorbidities on pregnancy (32). However, Black or African American infants continue to have higher rates of infant mortality due to PROM than any other race included in this study. This trend is consistent when stratified across regions and sex. This is consistent with current literature investigating Black or African American women’s infant mortality, maternal mortality, and obstetric complications (37–40).

The expansion of Medicaid through the ACA initiative was optional for states, leading to an unequal distribution of policy effects. It is estimated that the states which did not implement ACA expansion have nearly twice the uninsurance rate among rural residents (41). Therefore, implementation of ACA and CHIP may have initially reached larger cities disproportionately to rural areas, which may explain the significant decline in infant mortality in the larger metro areas after 2012. Additionally, the relatively higher rates of infant mortality in the metro areas compared to the nonmetro or small metro areas may also be explained by environmental factors such as air pollution. Dadvand et al. studied the association between air pollution exposure during the entire pregnancy and the last 3 months before rupture of membranes and found an increase in preterm PROM risk of up to 50% (3).

As PROM is an understudied cause of infant mortality, it is difficult to compare trends across countries. Therefore, to address population-based differences it may be helpful to examine overall infant mortality trends. Despite spending the largest amount on health-related goods and services, the United States ranked 33 out of 38 in infant mortality across all Organization for Economic Co-operation and Development (OECD) in 2021 (42). For reference, Japan and Norway held the lowest rates of 1.7 deaths per 1,000 live births, while the United States had 5.4 deaths per 1,000 live births across the same period (42). Additionally, in comparison to other OECD countries, the U.S. infant mortality has not improved to the same extent as that of other OECD countries (42).

Comparing infant mortality rates between the United States and Canada offers insight into potential pitfalls of the U.S. as Canada shares geographic proximity with contact between clinicians, acceptance and recognition of training for licensure in both countries, and a stark difference in healthcare systems. Despite the similarities, Canada continues to have a lower infant mortality rate than the U.S. (42, 43). In a comparison study, the U.S. was found to have higher rates of preterm birth and low birthweight, lower stillbirth rates at term, and substantially higher rates of late neonatal, post-neonatal, and infant mortality (43). It is postulated the lower preterm and low birthweight rates in Canada suggest better maternal health status and the increased post-neonatal survival suggest better infant care (43). In a different comparison study between the United States and European countries, the main cause of a higher infant mortality was a much higher percentage of preterm births (44). The increased health spending by the U.S. may benefit the early neonatal period survival due to advanced, aggressive early intervention, but it does not show the same effect after the early neonatal period (43). Additionally, it is postulated that differences in socio-demographic and behavioral factors attribute to the infant mortality rates. This is especially prevalent when taking into consideration that infant mortality rates overall were higher at every gestation week among the United States Black population (43). This is also reflected in our data with Black or African American population experiencing overall higher rates of infant mortality.

This study is limited by the CDC WONDER database, which may not represent or encompass all infants affected by PROM and their demographic characteristics. This study also utilized multiple cause mortality data, meaning that PROM is listed as a cause of death but may not be the sole contributing factor. Additionally, PROM and PPROM are not able to be distinguished in this study due to the limitations of ICD-10 codes available through CDC WONDER. Furthermore, this study examines infant mortality, which does not account for all diagnoses of PROM and therefore may miss risk factors for diagnosis. CDC WONDER also only accounts for liveborn infants affected by PROM, thereby excludes the fetal deaths due to PROM where no live birth occurs. Finally, infant race and maternal race are not always the same thus, inferences made through the lens of race may not exactly correlate with infant mortality rate data. Further studies are needed to examine individual factors contributing to PROM and PPROM separately.

The strength of this study is supported through the utilization of a nationally representative database. CDC WONDER database accounts for demographic factors associated with mortality data and allows for the intersectional analysis of such factors. Additionally, to our knowledge this is one of the first studies addressing infant demographic factors associated with PROM-related mortality as opposed to maternal factors.

## Conclusion

There was an overall decline in the infant mortality among newborns affected by premature rupture of membranes from 1999 to 2023. The most critical decline was seen after 2013, possibly reflecting the efficacy of the implementation of the Affordable Care Act and Children’s Health Insurance Program initiatives. However, this improvement was not seen equally across all demographic factors studied. Disparities by urban–rural residence and racial disparities are evident in the trends seen in this study, emphasizing the need for targeted healthcare initiatives and population-based research investigating risk factors and barriers faced by these populations.

Specifically, the ongoing discrepancy in infant mortality rate related to PROM between Black or African American and all other races studied should be investigated. Further research is needed to recognize and determine preventive factors for PROM and PPROM, and innovative interventions. As obstetric and NICU interventions advance and infant mortality declines, these demographic factors should be considered to ensure equitable reduction in PROM-related infant mortality.

## Data Availability

Publicly available datasets were analyzed in this study. This data can be found at: CDC WONDER Database [https://wonder.cdc.gov/mcd.html].
