# Supplementary material for: Epidemiological trends of infant mortality related to premature rupture of membranes: U.S. 1999–2023
Source: Front Public Health. 2026 Jan 12;13:1715138. doi: 10.3389/fpubh.2025.1715138 (PMC12832673; doi:10.3389/fpubh.2025.1715138)
Supplement: Supplementary file 1 [file Table_1.DOCX]

Supplementary Material

**
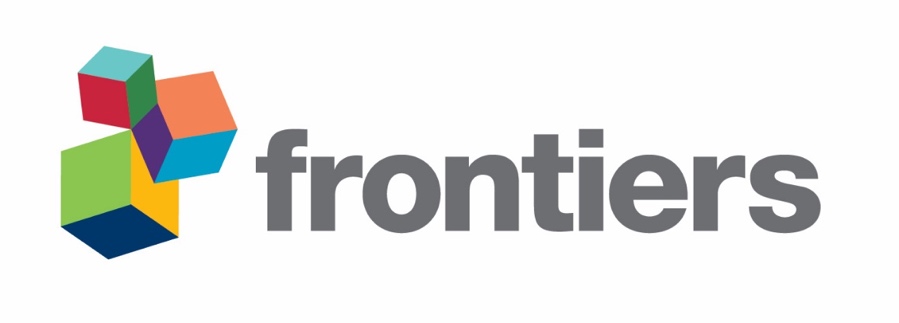
**

**Supplementary Figure 1.** Newborns Affected by Premature Rupture of Membranes Annual Percent Change Values for Investigated Variables

| **Demographic Factor** | **Cohort** | **Lower Endpoint** | **Upper Endpoint** | **APC** | **Lower CI** | **Upper CI** | **P-Value** |
| --- | --- | --- | --- | --- | --- | --- | --- |
| Overall | All | 1999 | 2002 | 5.0862* | 0.4081 | 14.6541 | 0.022795 |
|  |  | 2002 | 2013 | 0.3201 | -5.7142 | 1.6099 | 0.669466 |
|  |  | 2013 | 2023 | -2.098 | -7.1972 | 1.6369 | 0.094781 |
| Gender | Female | 1999 | 2005 | 2.9667* | 0.1346 | 13.1077 | 0.037193 |
|  |  | 2005 | 2023 | -1.0555* | -3.2032 | -0.5253 | 0.0024 |
|  | Male | 1999 | 2011 | 1.4169* | 0.6968 | 2.4537 | 0.0004 |
|  |  | 2011 | 2023 | -2.1606* | -3.2034 | -1.4491 | < 0.000001 |
| Urban/Rural | Large Central Metro | 1999 | 2012 | 2.1393* | 1.0063 | 4.5503 | 0.0004 |
|  |  | 2012 | 2021 | -2.9272* | -7.6212 | -0.9363 | 0.0016 |
|  | Large Fringe Metro | 1999 | 2012 | 1.4501* | 0.4246 | 3.1901 | 0.009598 |
|  |  | 2012 | 2021 | -3.9319* | -6.3049 | -2.4367 | < 0.000001 |
|  | Medium Metro | 1999 | 2002 | 8.4597* | 0.9643 | 23.1007 | 0.015597 |
|  |  | 2002 | 2021 | -0.8825* | -1.9021 | -0.404 | 0.002799 |
|  | Small Metro | 1999 | 2021 | -1.0421 | -2.1449 | 0.025 | 0.053989 |
|  | Micropolitan (Nonmetro) | 1999 | 2021 | -1.0039* | -2.0269 | -0.0067 | 0.04759 |
|  | NonCore (Nonmetro) | 1999 | 2021 | -1.3567* | -2.5883 | -0.286 | 0.017197 |
| Region | Northeast | 1999 | 2006 | 3.227 | -0.6994 | 30.2143 | 0.120376 |
|  |  | 2006 | 2023 | -2.3107* | -16.0199 | -1.2462 | 0.007598 |
|  | Midwest | 1999 | 2023 | -0.7504* | -1.3883 | -0.1417 | 0.013597 |
|  | South | 1999 | 2004 | 2.9546* | 0.1069 | 10.1986 | 0.041592 |
|  |  | 2004 | 2023 | -1.3788* | -2.2568 | -0.9812 | < 0.000001 |
|  | West | 1999 | 2009 | 4.5745* | 2.7469 | 9.9202 | < 0.000001 |
|  |  | 2009 | 2023 | -0.6244 | -3.6082 | 0.769 | 0.369526 |
| Race | Asian or Pacific Islander | 1999 | 2023 | 1.9680* | 0.5734 | 3.5891 | 0.007998 |
|  | Black or African American | 1999 | 2005 | 2.5755 | -0.4448 | 12.3089 | 0.091582 |
|  |  | 2005 | 2023 | -2.3156* | -3.7895 | -1.709 | < 0.000001 |
|  | White | 1999 | 2010 | 1.0155* | 0.0567 | 2.4264 | 0.037193 |
|  |  | 2010 | 2023 | -3.1062* | -4.3422 | -2.3132 | < 0.000001 |
|  | Hispanic or Latino | 1999 | 2005 | 5.5813* | 1.7375 | 21.1035 | < 0.000001 |
|  |  | 2005 | 2023 | 0.4488 | -2.0432 | 1.1187 | 0.428714 |
| Gender + Race | Asian or Pacific Islander / Female | 2012 | 2022 | 0.1829 | -3.7693 | 3.8112 | 0.867427 |
|  | Asian or Pacific Islander / Male | 2007 | 2022 | 0.9332 | -2.7969 | 4.8429 | 0.535893 |
|  | Black or African American / Female | 1999 | 2005 | 2.5688 | -1.2665 | 17.0901 | 0.20036 |
|  |  | 2005 | 2023 | -2.6916* | -6.8636 | -1.9064 | 0.003599 |
|  | Black or African American / Male | 1999 | 2006 | 2.0205 | -0.4554 | 9.8654 | 0.09958 |
|  |  | 2006 | 2023 | -2.1610* | -3.9421 | -1.4901 | 0.0008 |
|  | White / Female | 1999 | 2002 | 8.6303 | -0.8167 | 33.0863 | 0.109178 |
|  |  | 2002 | 2023 | -1.5599* | -9.3661 | -0.9544 | 0.014397 |
|  | White / Male | 1999 | 2011 | 0.9409 | -0.1997 | 2.822 | 0.09918 |
|  |  | 2011 | 2023 | -3.8156* | -6.0521 | -2.5847 | < 0.000001 |
|  | Hispanic or Latino / Female | 2000 | 2023 | 1.1108 | -0.0149 | 2.2631 | 0.05199 |
|  | Hispanic or Latino / Male | 1999 | 2005 | 5.8364* | 1.6906 | 24.6101 | < 0.000001 |
|  |  | 2005 | 2023 | 0.3412 | -2.5769 | 1.0614 | 0.616277 |
| Gender + Region | Female / Northeast | 1999 | 2023 | -1.1095 | -2.2623 | 0.0249 | 0.055989 |
|  | Female / Midwest | 1999 | 2011 | 0.4402 | -0.6435 | 7.5497 | 0.422316 |
|  |  | 2011 | 2023 | -2.1520* | -9.4213 | -1.0235 | 0.004399 |
|  | Female / South | 1999 | 2023 | -0.8807* | -1.4916 | -0.3311 | 0.0016 |
|  | Female / West | 1999 | 2009 | 4.5918* | 2.0439 | 21.3266 | < 0.000001 |
|  |  | 2009 | 2023 | -0.3275 | -5.3696 | 0.9714 | 0.520696 |
|  | Male / Northeast | 1999 | 2006 | 4.1601* | 0.6476 | 17.4319 | 0.025595 |
|  |  | 2006 | 2023 | -3.0489* | -5.3227 | -1.9617 | < 0.000001 |
|  | Male / Midwest | 1999 | 2023 | -0.3957 | -1.1565 | 0.299 | 0.242352 |
|  | Male / South | 1999 | 2008 | 1.7173* | 0.3847 | 4.2117 | 0.011198 |
|  |  | 2008 | 2023 | -2.0006* | -3.0716 | -1.3542 | < 0.000001 |
|  | Male / West | 1999 | 2010 | 4.6394* | 1.896 | 24.4781 | 0.0024 |
|  |  | 2010 | 2023 | -1.3138 | -8.9154 | 0.5722 | 0.144371 |
| Gender + Urban / Rural | Large Central Metro / Female | 1999 | 2005 | 4.4853* | 0.5351 | 19.4296 | 0.018396 |
|  |  | 2005 | 2021 | -0.8341 | -6.447 | 0.0133 | 0.05239 |
|  | Large Central Metro / Male | 1999 | 2012 | 2.8542* | 1.4018 | 7.5602 | < 0.000001 |
|  |  | 2012 | 2021 | -3.0687* | -10.0814 | -0.5178 | 0.013997 |
|  | Large Fringe Metro / Female | 1999 | 2005 | 4.0274* | 0.1666 | 21.3861 | 0.034393 |
|  |  | 2005 | 2021 | -1.2035* | -8.0308 | -0.2666 | 0.020796 |
|  | Large Fringe Metro / Male | 1999 | 2015 | 0.7664 | -0.1205 | 2.4099 | 0.085183 |
|  |  | 2015 | 2021 | -6.3196* | -16.0567 | -2.56 | < 0.000001 |
|  | Medium Metro / Female | 1999 | 2021 | -0.5017 | -1.8706 | 0.84 | 0.435113 |
|  | Medium Metro / Male | 1999 | 2006 | 3.7721* | 0.9768 | 13.2832 | 0.004799 |
|  |  | 2006 | 2021 | -1.4485* | -4.1907 | -0.5172 | 0.002 |
|  | Small Metro / Female | 1999 | 2021 | -1.7391* | -3.3613 | -0.2636 | 0.023195 |
|  | Small Metro / Male | 1999 | 2021 | -0.334 | -1.4244 | 0.7167 | 0.525095 |
|  | Micropolitan (Nonmetro) / Female | 1999 | 2021 | -0.014 | -1.2916 | 1.2891 | 0.993001 |
|  | Micropolitan (Nonmetro) / Male | 1999 | 2021 | -1.4925* | -2.5875 | -0.4551 | 0.004399 |
| Region + Race | Northeast / Black or African American | 1999 | 2004 | 8.1225 | -0.4227 | 47.1637 | 0.067986 |
|  |  | 2004 | 2023 | -2.5231* | -10.592 | -1.4257 | 0.003999 |
|  | Midwest / Black or African American | 1999 | 2023 | -0.6704 | -2.4001 | 1.0653 | 0.365527 |
|  | South / Black or African American | 1999 | 2005 | 2.1544 | -0.6637 | 10.7272 | 0.130374 |
|  |  | 2005 | 2023 | -2.9490* | -4.1253 | -2.3594 | < 0.000001 |
|  | West / Black or African American | 2000 | 2020 | 0.2698 | -1.4762 | 2.1558 | 0.721856 |
|  | Northeast / White | 1999 | 2013 | 0.6137 | -0.6446 | 2.2578 | 0.332334 |
|  |  | 2013 | 2023 | -7.3682* | -11.5127 | -5.0715 | < 0.000001 |
|  | Midwest / White | 1999 | 2023 | -1.6765* | -2.3827 | -1.0344 | < 0.000001 |
|  | South / White | 1999 | 2002 | 7.5593 | -1.0545 | 28.0413 | 0.153569 |
|  |  | 2002 | 2023 | -1.4901* | -10.816 | -0.8189 | 0.027195 |
|  | West / White | 1999 | 2005 | 9.2268* | 0.9737 | 45.32 | 0.012398 |
|  |  | 2005 | 2023 | -1.4958* | -12.7431 | -0.027 | 0.04839 |
|  | Hispanic or Latino / Northeast | 2001 | 2023 | -0.9119 | -1.9651 | 0.159 | 0.091582 |
|  | Hispanic or Latino / Midwest | 2004 | 2023 | 0.6469 | -1.2169 | 2.5674 | 0.465507 |
|  | Hispanic or Latino / South | 1999 | 2023 | 1.7157* | 0.6187 | 3.0027 | 0.004399 |
|  | Hispanic or Latino / West | 1999 | 2008 | 4.7530* | 2.2195 | 16.1326 | 0.0008 |
|  |  | 2008 | 2023 | -0.031 | -3.5306 | 1.0526 | 0.80024 |
